# Establishment of an Endocytosis-Related Prognostic Signature for Patients With Low-Grade Glioma

**DOI:** 10.3389/fgene.2021.709666

**Published:** 2021-09-06

**Authors:** Dawei Wang, Shiguang Liu, Guangxin Wang

**Affiliations:** ^1^Shandong Academy of Clinical Medicine, Provincial Hospital Affiliated to Shandong First Medical University, Jinan, China; ^2^Shandong Academy of Clinical Medicine, Shandong Provincial Hospital, Cheeloo College of Medicine, Shandong University, Jinan, China; ^3^Research Center of Translational Medicine, Jinan Central Hospital, Cheeloo College of Medicine, Shandong University, Jinan, China; ^4^Shandong Innovation Center of Intelligent Diagnosis, Central Hospital Affiliated to Shandong First Medical University, Jinan, China

**Keywords:** low-grade glioma, biomarker, endocytosis-related gene, model, prognostic signature

## Abstract

**Background:**

Low-grade glioma (LGG) is a heterogeneous tumor that might develop into high-grade malignant glioma, which markedly reduces patient survival time. Endocytosis is a cellular process responsible for the internalization of cell surface proteins or external materials into the cytosol. Dysregulated endocytic pathways have been linked to all steps of oncogenesis, from initial transformation to late invasion and metastasis. However, endocytosis-related gene (ERG) signatures have not been used to study the correlations between endocytosis and prognosis in cancer. Therefore, it is essential to develop a prognostic model for LGG based on the expression profiles of ERGs.

**Methods:**

The Cancer Genome Atlas and the Genotype-Tissue Expression database were used to identify differentially expressed ERGs in LGG patients. Gene ontology, Kyoto Encyclopedia of Genes and Genomes, and Gene set enrichment analysis methodologies were adopted for functional analysis. A protein-protein interaction (PPI) network was constructed and hub genes were identified based on the Search Tool for the Retrieval of Interacting Proteins database. Univariate and multivariate Cox regression analyses were used to develop an ERG signature to predict the overall survival (OS) of LGG patients. Finally, the association between the ERG signature and gene mutation status was further analyzed.

**Results:**

Sixty-two ERGs showed distinct mRNA expression patterns between normal brain tissues and LGG tissues. Functional analysis indicated that these ERGs were strikingly enriched in endosomal trafficking pathways. The PPI network indicated that EGFR was the most central protein. We then built a 29-gene signature, dividing patients into high-risk and low-risk groups with significantly different OS times. The prognostic performance of the 29-gene signature was validated in another LGG cohort. Additionally, we found that the mutation scores calculated based on the *TTN*, *PIK3CA*, *NF1*, and *IDH1* mutation status were significantly correlated with the endocytosis-related prognostic signature. Finally, a clinical nomogram with a concordance index of 0.881 predicted the survival probability of LGG patients by integrating clinicopathologic features and ERG signatures.

**Conclusion:**

Our ERG-based prediction models could serve as an independent prognostic tool to accurately predict the outcomes of LGG.

## Introduction

Glioma is the most common malignant tumor in the central nervous system among histological subtypes of brain tumors (Louis et al., 2007). Gliomas are classified into four grades based on their clinical and histopathological characteristics (Louis et al., 2016; Barnholtz-Sloan et al., 2018). Grade I and Grade II gliomas are regarded as low-grade gliomas (LGGs) and include pilocytic astrocytomas, diffuse astrocytomas, and oligodendrogliomas; Grade III and Grade VI gliomas are considered as high-grade gliomas and include anaplastic astrocytomas, anaplastic oligodendrogliomas, and glioblastoma multiforme (GBM). GBM is the most frequent cancer in the adult brain, with a 5-year survival rate of less than 10% (Preusser et al., 2011; Ostrom et al., 2018). LGG is considered a comparatively benign tumor. It has a 5-year survival rate of 59.9% (Claus and Black, 2006). However, 70% of LGG patients are likely to develop GBM within 5–10 years (Furnari et al., 2007). Currently, gliomas are primarily detected based on pathological features or by imaging-based methods such as CT, MRI, and PET, etc., the reliability of which is mainly dependent on the surgeon’s experience (Mörén et al., 2015; Yang et al., 2019). These tests or examinations may not detect gliomas initially; thus, the chance to use surgical treatment strategies is missed, leading to more patient deaths. At the molecular level, O-6-methylguanine- DNA methyltransferase promoter methylation, *EGFR* alterations, *IDHI or IDH2* mutation, and 1p19q codeletion are tested to diagnose gliomas (Rodriguez et al., 2016). However, these markers are less sensitive and specific for detecting gliomas at an early stage. Thus, it is imperative to discover effective diagnostic and prognostic models to detect and predict the prognosis of LGG.

Endocytosis is an evolutionarily conserved cellular process by which molecules are actively transported into the cell via engulfment with the membrane (Doherty and McMahon, 2009). Endocytic cargo, including extracellular molecules, plasma membrane proteins, or membrane lipids, can be internalized through the eukaryotic cell surface by a clathrin-dependent or clathrin-independent process (Mayor and Pagano, 2007; McMahon and Boucrot, 2011). The endosomal trafficking system comprises a series of dynamically interconnected membrane-enclosed vesicular structures, including early endosomes, recycling endosomes, and late endosomes, which are vital for maintaining cellular homeostasis and energy recycling (Di Fiore and von Zastrow, 2014; Scott et al., 2014). Once inside cells, these vesicles are subjected to various homotypic fusion events to form early endosomes, which can act as the primary sorting hub. For example, essential receptors can be recycled back from early endosomes to the plasma membrane, whereas others can be transported to the *trans*-Golgi network or late endosomes; late endosomes would fuse with lysosomes to form endolysosomes where most of the endosomal cargoes are degraded (Di Fiore and von Zastrow, 2014; Scott et al., 2014). The maturation, sorting, and trafficking events of these vesicles are precisely controlled by RAB small GTPases and their effectors and membrane tethering complexes, as well as by phosphatidylinositol phospholipids and their catalyzing enzymes (Pfeffer, 2017; Wallroth and Haucke, 2018; Ungermann and Kümmel, 2019). However, dysfunction of these regulators results in a dysregulated endocytosis phenotype, which has emerged as a multifaceted cancer cell hallmark (Mosesson et al., 2008). In addition, cancer cells undergo constitutive endocytosis more rapidly than the paired noncancerous cells, thereby providing more nutrients and signaling support (Mellman and Yarden, 2013). For example, cancer cell growth is regulated by growth hormone receptors, whose amounts are controlled by endocytosis (Mellman and Yarden, 2013); activated receptor tyrosine kinases (RTK)-ligand complexes, e.g., EGFR-EGF, could activate its downstream targets, such as MEK–ERK pathway, to promote cell growth (Roberts and Der, 2007). Interestingly, their internalization from the cell surface and degradation in the lysosomes are controlled by endocytosis (Goh and Sorkin, 2013); therefore, abnormal endocytosis could influence cell survival and proliferation via affecting the initiation and termination of the RTK related signal cascades.

There are many signaling pathways, such as autophagy, immune microenvironment, and epigenetic modifications, involved in tumorigenesis and development. Several papers had already established the association of those signalings with the LGG prognosis (Tu et al., 2020; Zhang et al., 2020; Chen et al., 2021). Although numerous studies have reported the relationship of endocytosis with cancer development, prognostic models for endocytosis-related genes (ERGs) in cancer have not been investigated. Since the dysregulation of the endocytosis pathway or its regulatory genes has been observed in LGG (Zhou et al., 2017; Liu et al., 2020), and some ERGs have exhibited their potential to predict the OS of LGG patients (An et al., 2020); therefore, it is reasonable to suppose that ERGs hold great promise for predicting the prognosis of LGG. In this work, we identified the differential ERGs between LGG samples and matched normal brain samples by analyzing high-throughput mRNA data downloaded from The Cancer Genome Atlas (TCGA) and the Genotype-Tissue Expression (GTEx) database. Gene ontology (GO) functional and Kyoto Encyclopedia of Genes and Genomes (KEGG) pathway analyses, as well as PPI network analysis, were performed with the differential ERGs. Additionally, we established an endocytosis-related risk signature that contains 29 ERGs, which can be used to evaluate the prognosis of LGG patients. Moreover, we identified the ten most frequently mutated genes identified in LGG patients and found that *TTN*, *PIK3CA*, *NF1*, and *IDH1* based mutation scores could predict prognosis and were positively correlated with the endocytosis-related prognostic signature. Finally, a clinical nomogram with a concordance index of 0.881 was constructed by combining clinicopathologic features and ERG signatures to predict the survival probability of LGG patients.

## Materials and Methods

### Endocytosis-Related Gene Set

Endocytosis-related genes were retrieved by searching the GeneCards website^1^ with the term “endosome.” A relevance score ranging from 0 to 100 was used to indicate the correlation between genes and endosomal activity. Higher scores indicated stronger associations. Genes with an association score >1 were collected; after manual filtering, 676 genes were finally included in the ERGs set.

### Acquisition of Patient Samples

The GTEx and TCGA gene expression data, which contain 1,152 normal tissue samples and 523 tumor samples, respectively, were downloaded from UCSC Toil RNAseq Recompute Compendium^2^, in which all raw RNA-Seq data are re-computed based on a uniform pipeline (Vivian et al., 2017; Goldman et al., 2020). The mRNA-array_301 dataset, which comprises 156 LGG patients’ samples, was obtained from the Chinese Glioma Genome Atlas (CGGA)^3^ for validation studies.

### Differentially Expressed ERGs and Functional Enrichment

The differential expression of ERGs between LGG and normal brain tissues was analyzed using the limma package in R, with the following thresholds: |log_2_ fold change (FC)**| >** 1 and adjusted *P*-value < 0.05. Then, GO enrichment analysis was performed to identify the significant biological attributes of differentially expressed ERGs (DEERGs) by using the “ggplot2” and “enrichplot” packages in R.

### Construction and Analysis of the PPI Network

The Search Tool for the Retrieval of Interacting Proteins (STRING) online database^4^ and Cytoscape software^5^ were used to identify hub genes and visualize the protein-protein interactions (PPIs) of the DEERGs, respectively.

### Gene Set Enrichment Analysis

Gene set enrichment analysis (GSEA) was performed using GSEA software version 3.0^6^. Single-gene GSEA was performed to identify the significant pathways between the low EGFR expression and high EGFR expression groups of TCGA-LGG patients, containing 529 samples. The FPKM data was downloaded from GDC Xena Hub^7^. GSEA was also used to predict differences in the biological processes between the high-risk and low-risk LGG patient groups.

### Gene Mutation Query

The mutation status and survival outcome of interesting genes were searched on the cBioportal database^8^. The cBioportal is an open-access resource to analyze and visualize cancer genomics data integrated from different studies, e.g., TCGA. Specifically, we selected the Firehose Legacy, Pan Cancer Atlas, and UCSF cohorts in the CNS/Brain category, which totally contains 1,105 samples, to analyze genes of interest in LGG patients.

### Construction and Validation of the Prognostic Gene Signature

Univariate Cox regression analysis was used to select candidate prognostic DEERGs that were significantly correlated with OS. Multivariate Cox regression analysis was performed to fit the DEERGs. The risk score for each patient was calculated with the estimated regression coefficient as the weight. The risk score for each patient was calculated as follows: risk score = $\sum_{i=1}^{n}\text{Coef}\,i$ × (multiply) EXP gene_(i)_, with Coef *i* indicating the Cox regression coefficient of gene*i* and EXP gene_(i)_ representing the relative expression level of each ERG. The median risk score was chosen as the cutoff value to dichotomize the TCGA-LGG cohort. The sensitivity and specificity of the risk score-based prediction models were compared using time-dependent receiver operating characteristic (ROC) curves. Kaplan–Meier survival analysis and log-rank tests were performed to compare survival differences. Multivariate analyses were performed using Cox proportional hazards regression analyses to assess whether the endocytosis gene signature was independent of other clinicopathological factors. The prognostic gene signature was verified mRNA-array_301 cohort, which the same formula used for the TCGA dataset was applied.

### Construction of the Nomogram

The nomogram was plotted based on age, gene mutation status, and risk score with the survival and RMS packages in R. Calibration curves were then generated to compare the predicted survival and actual outcomes. Moreover, the concordance index (C-index), ranging from 0.5 to 1.0, was computed to assess the prognostic model’s performance. Values of 0.5 and 1.0 represent a random guess and perfect prediction, respectively, for predicting survival with the model.

### Statistical Analysis

All statistical analyses were done with R and GraphPad Prism software. We provided R code in GitHub^9^ to reproduce our study. A *P*-value of less than 0.05 was considered to be statistically significant for all analyses.

## Results

### Distinctly Expressed ERGs in Normal Brain and Lower-Grade Glioma Tissues

**FIGURE 1:**
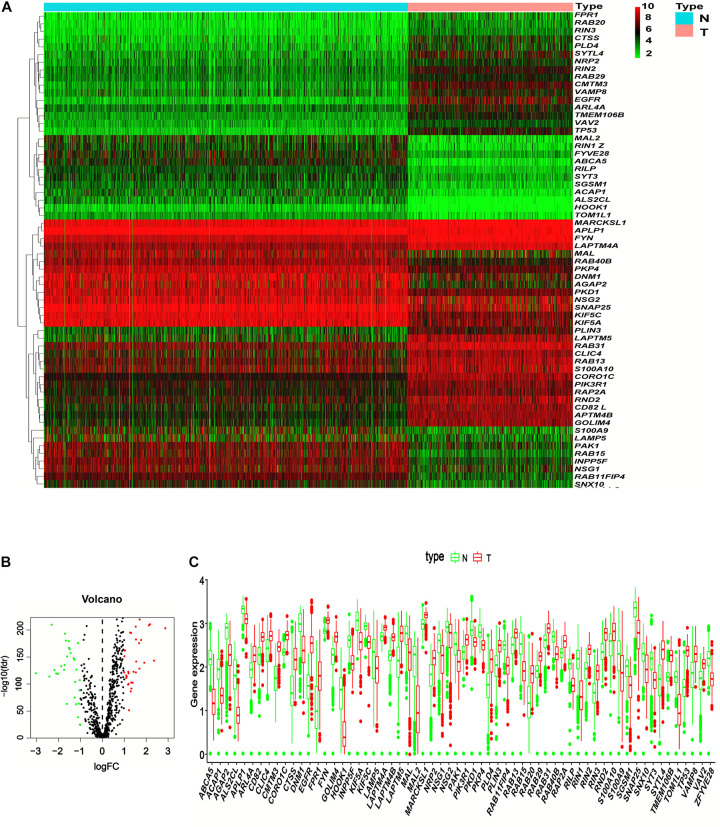
Identification of differentially expressed endocytosis-related genes (DEERGs) between LGG and normal brain tissue. **(A)** 62 DEERGs were plotted in the heatmap. Columns represent the different samples, and rows represent genes. The gene marked with red color indicates upregulation, whereas the green color indicates downregulation. **(B)** The volcano plot of the differential expression of 676 endocytosis-related genes (ERGs) between LGG and normal brain tissue. The red and green colors showed highly expressed or low expressed genes, respectively. **(C)** The expression levels of 62 DEERGs were shown in the boxplot. N, normal brain tissue; T, LGG. The DEERGs were filtered with the threshold of |log_2_ fold change (FC)| > 1 and FDR < 0.05.

In the present study, a total of 1,675 samples, comprising 1,152 normal patient samples, and 523 tumor samples, were included. Then, a total of 676 ERGs were obtained after manually filtering the genes with a relevance score > 1 (Supplementary Table 1). We identified 62 differential ERGs—32 up-regulated and 30 down-regulated ERGs—with the thresholds of |log_2_ fold change (FC)| > 1 (Figure 1A). Then, the expression patterns of DEERGs between LGG and nontumor tissues were visualized in a volcano plot and box plot (Figures 1B,C).

### Construction of the DEERG Regulatory Network

**FIGURE 2:**
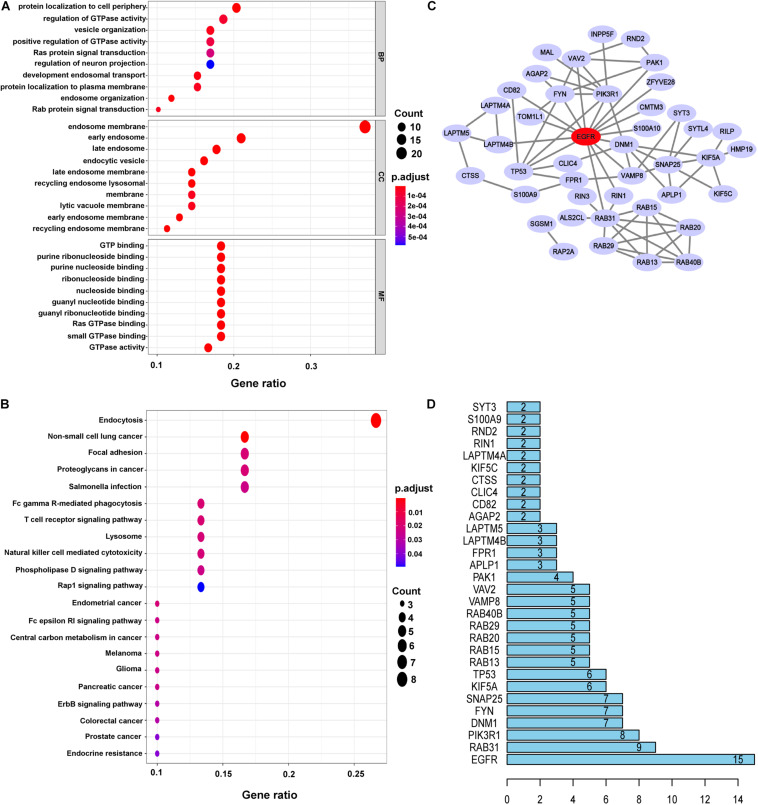
Functional enrichment analysis and construction PPI networks of DEERGs. **(A)** GO analysis of DEERGs. **(B)** KEGG analysis of DEERGs. **(C)** Cytoscape constructed PPI networks of DEERGs. **(D)** Identification of EGFR as the central protein.

To gain more biological insight into these DEERGs, GO enrichment analysis was performed. The DEERGs were classified into three functional groups: biological process (BP), cellular component (CC), and molecular function (MF). As shown in Figure 2A and Supplementary Table 2, the top enriched GO terms for BP were protein localization to the cell periphery, regulation of GTPase activity, and vesicle organization; in the CC category, the DEERGs were potentially localized at the endosome membrane, early endosome, and late endosome; and in the MF category, GTP binding, and purine ribonucleoside binding, and purine nucleotide binding were the most significantly enriched terms. Interestingly, after removing the common GO terms from over-, under-, and non-differentially expressed genes, we can obtain several specific GO terms of DEERGs, e.g., positive regulation of intracellular protein transport in BP, lateral plasma membrane in CC, and structural constituent of myelin sheath in MF (Supplementary Figure 1). Further KEGG analysis showed that the DEERGs were significantly associated with endocytosis, non-small cell lung cancer, and focal adhesion pathways (Figure 2B and Supplementary Table 3). Similarly, after removing the common enriched pathways in over-, under-, and non-differentially expressed genes, we found that non-small cell lung cancer, and focal adhesion, and T cell receptor signaling pathways could be specifically enriched by DEERGs.

**FIGURE 3:**
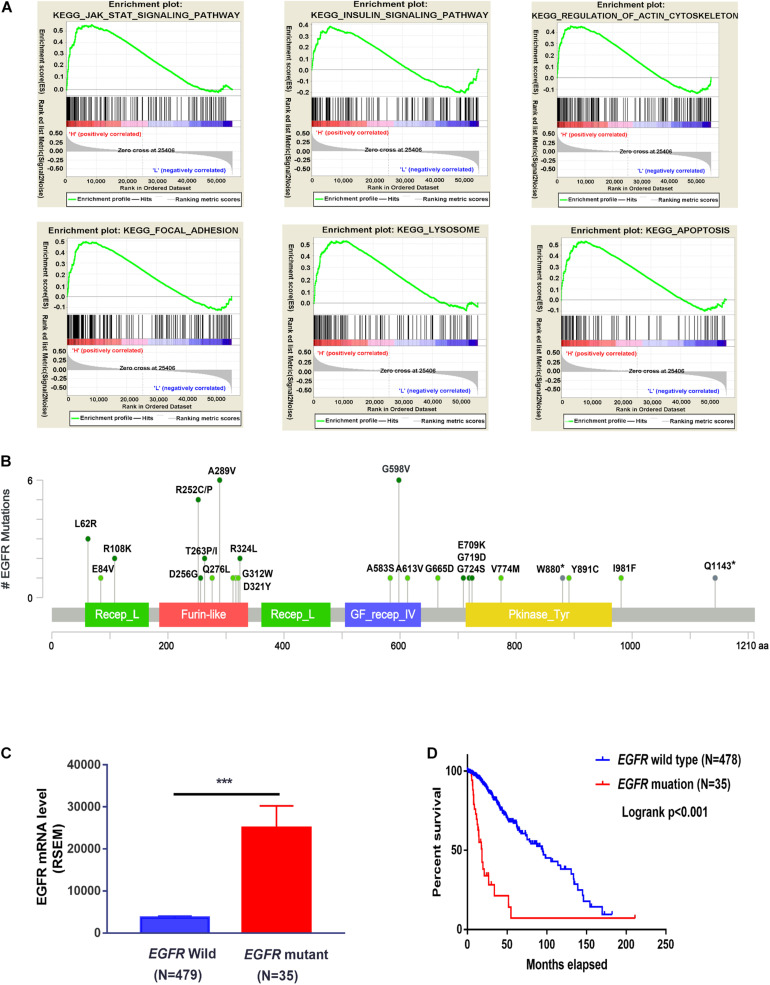
Functional analysis of *EGFR* in LGG cohorts. **(A)** GSEA analysis of *EGFR* in the TCGA-LGG cohort. **(B)** *EGFR* mutation sites were analyzed three LGG cohorts from cBioportal. **(C)** The expression difference of wild-type *EGFR* and mutated *EGFR* in TCGA-LGG patients (TCGA, PanCancer Atlas). **(D)** The survival probability of LGG patients with or without *EGFR* mutation (TCGA, PanCancer Atlas). ***P < 0.001.

The PPI network of the DEERGs was constructed and visualized via the STRING online database and Cytoscape software, respectively. Based on the results shown in Figures 2C,D, EGFR was considered a central hub protein connected to fifteen nodes. To better understand the biological contribution of *EGFR*, single-gene GSEA was performed. As shown in Figure 3A, *EGFR* was significantly positively related to six KEGG pathways: the Jak-STAT signaling, insulin signaling, regulation of actin cytoskeleton, focal adhesion, lysosome, and apoptosis pathways. Furthermore, we found that there were many *EGFR* mutation types in LGG patients (Figure 3B), and most of these mutations were oncogenic. More significantly, the expression levels of these *EGFR* mutant forms were higher than that of wild-type *EGFR* in LGG tissues, and patients harboring those *EGFR* mutations showed much lower OS rates (Figures 3C,D).

### Construction of the Prognostic Signature for the TCGA-LGG Cohort

**FIGURE 4:**
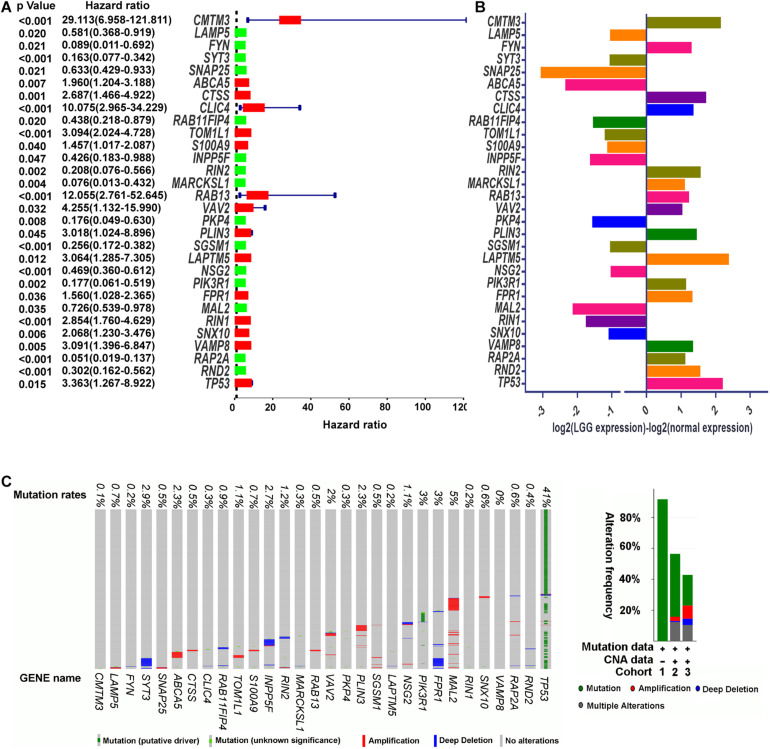
Identification of prognosis-related DEERGs. **(A)** Univariate Cox proportional hazard regression analysis of DEERGs. **(B)** Comparison of prognosis-related DEERGs mRNA expression between normal brain tissues and TCGA-LGG tissues. **(C)** Genetic alteration of prognosis-related DEERGs in LGG sample. Cohort 1: Low-Grade Gliomas (Johnson et al., 2014); Cohort 2: Brain Lower Grade Glioma (TCGA, PanCancer Atlas); and Cohort 3: Brain Lower Grade Glioma (TCGA, Firehose Legacy). Hazard ratio estimates the ratio of the hazard rate under two levels of an explanatory variable, such as female VS male. The hazard rate is the probability of an event occurring per unit of time.

To investigate whether the DEERGs could be used for prognosis prediction in LGG patients, univariate Cox proportional hazards regression analysis was performed with each DEERG using the expression profiles in the TCGA-LGG cohort. As shown in Figures 4A,B, a total of 30 genes were significantly associated with survival in the TCGA-LGG cohort, with 14 down-regulated and the other 16 up-regulated. To examine whether the genomic alterations in these risk-associated genes in LGG contribute to brain carcinogenesis, 1,105 LGG patient samples in cBioportal database, including both mutation and copy number alteration data, were analyzed. As shown in Figure 4C, genes of interest were altered in 533 (51%) of the 1,105 queried patients/samples, and this high genetic alteration rate indicated the crucial roles of these genes in the development of LGG.

**FIGURE 5:**
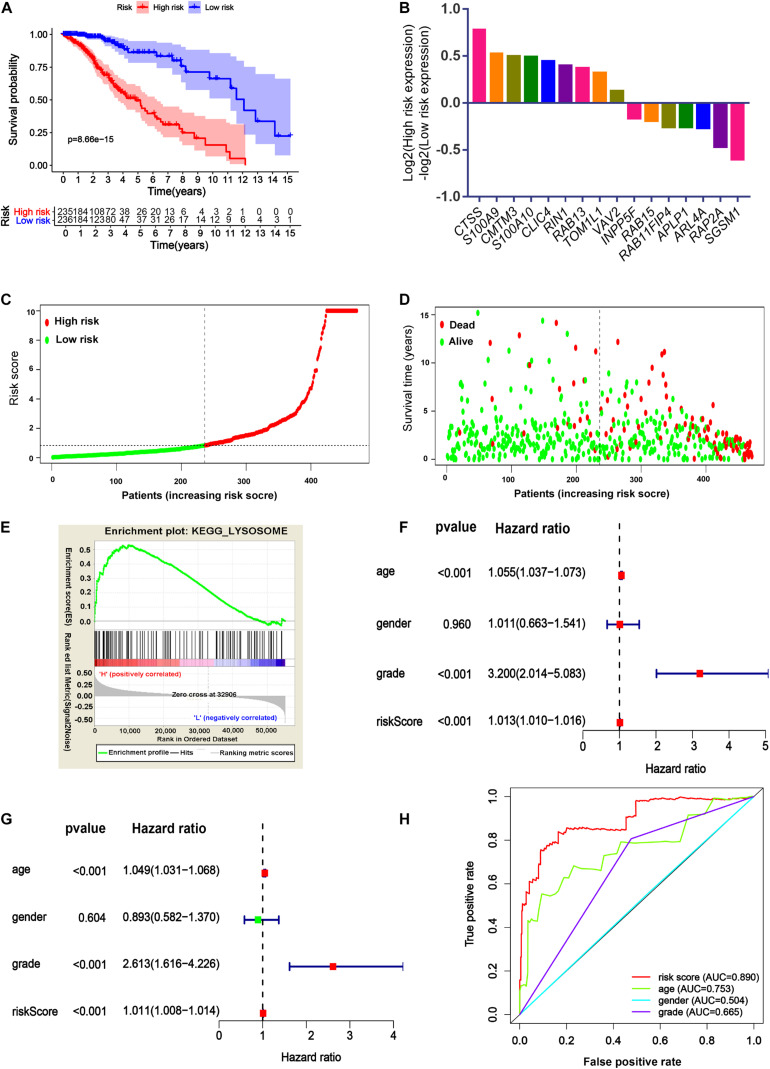
Identification of 29-ERGs signature for the prognosis of TCGA-LGG cohort. **(A)** Kaplan–Meier analysis of the overall survival of low-risk and high-risk LGG patients stratified by the median risk score. **(B)** The expression of 16- DEERGs in low-risk and high-risk LGG patients. **(C,D)** The distribution of risk score and patients’ survival status between low- and high-risk groups. **(E)** GSEA analysis of KEGG pathway enriched in the high-risk groups. **(F)** Univariate Cox regression analysis **(G)** Multivariate Cox regression analysis. **(H)** Muti-index of ROC curve.

To identify the best model for predicting patient prognosis, multivariate Cox proportional hazards regression analysis was further carried out on all 62 DEERGs, which finally identified 29 genes. The risk score to predict OS for each patient was calculated as described in the materials and methods section. Based on the risk score, 471 LGG patients from the TCGA database were classified into a low-risk and a high-risk group. Kaplan–Meier survival curves indicated that the OS time of low-risk patients was significantly longer than that of high-risk patients (Figure 5A). As shown in Figure 5B, among the above 29 genes, *CTSS*, *S100A9*, *CMTM3*, *S100A10*, *CLIC4*, *RIN1*, *RAB13*, *TOM1L1*, and *VAV2* were significantly up-regulated in the high-risk group, whereas *INPP5F*, *RAB15*, *RAB11FIP4*, *APLP1*, *ARL4A*, *RAP2A*, and *SGSM1* were significantly down-regulated. The distributions of risk score and survival status in each patient were also analyzed, as illustrated in Figures 5C,D. In addition, GSEA was performed based on each patient’s risk score; as presented in Figure 5E and Supplementary Table 4, the lysosome pathway was one of the highest enriched pathways in patients with high-risk scores, indicating that the dysregulation of lysosomes might affect LGG progression.

Univariate and multivariate models were built to analyze the independent prognostic value of different factors, such as age, sex, grade, and risk score, in LGG. The results suggested that age, grade, and risk score were independent prognostic indicators (Figures 5F,G). Further ROC curve analysis demonstrated that the risk score, with the highest area under the curve (AUC) value (0.89), had the best prognostic performance among the prognostic indicators (Figure 5H).

### Validation of the Prognostic Gene Signature in an LGG Cohort From the CGGA Database

**FIGURE 6:**
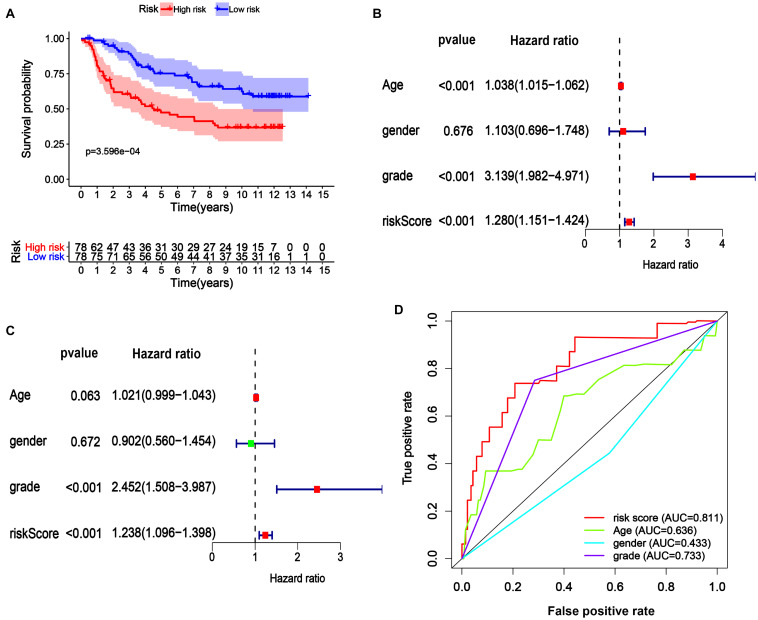
Verification of 29-ERGs signature for the prognosis of a CGGA-LGG cohort. The Kaplan-Meier analysis **(A)**, Univariate Cox regression analysis **(B)**, and Multivariate Cox regression analysis **(C)**, and Muti-index of ROC curve **(D)** for the mRNA-array_301 cohort.

We next assessed the prognostic gene signature’s predictive power in another LGG cohort (mRNA-array_301) from the CGGA database. In each cohort, patients were divided into a low-risk and a high-risk group based on the calculated risk score before comparing OS between the two groups. As expected, the survival curves in Figure 6A indicate that in the dataset mRNA-array_301, which included 157 patients, the OS time was much lower in the high-risk group than in the low-risk group (median OS time = 3.65 years vs. 8.58 years, *P* < 0.001). Univariate and multivariate Cox regression analyses indicated that the risk score could be an independent prognostic indicator in the LGG cohort (Figures 6B,C). More significantly, as shown in Figure 6D, the AUC of the risk score was higher than that of age, sex, and grade in the LGG cohort, further confirming the excellent power of this 29-endocytosis-related-gene signature as an independent prognostic predictor for LGG.

### Nomogram Development for Personalized Prognosis Prediction

**FIGURE 7:**
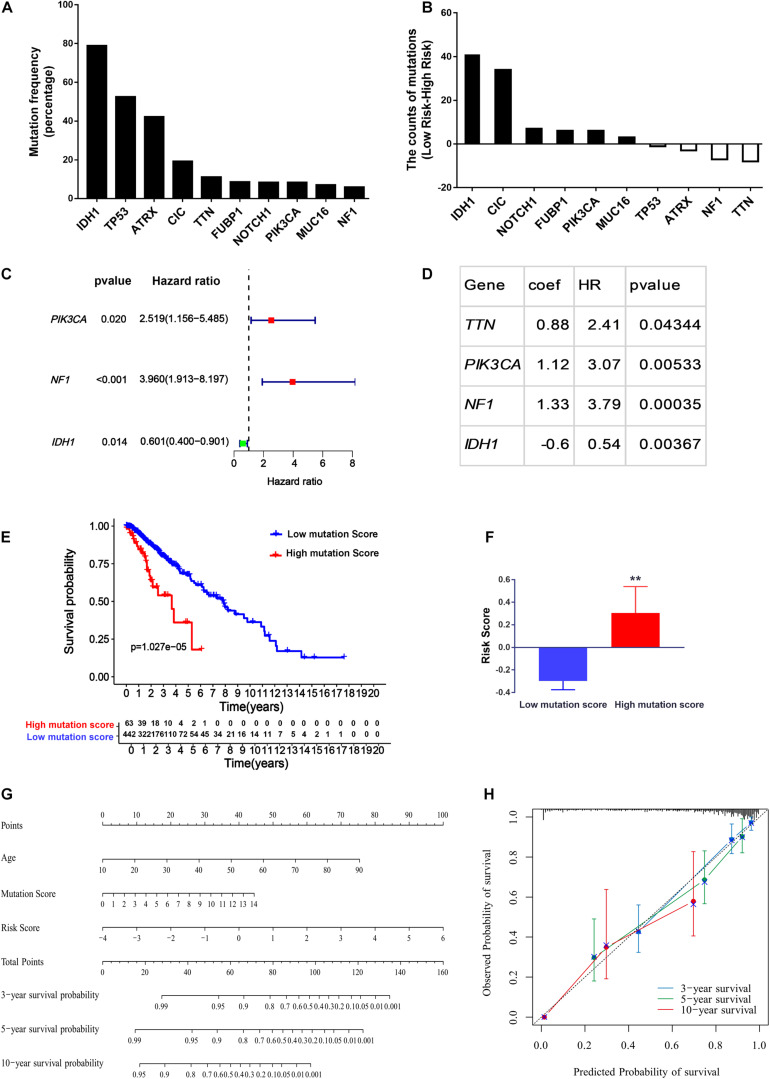
Construction of the nomogram for predicting the overall survival of LGG patients. **(A)** Top ten genes with the highest mutation rates in the LGG patients obtained from cBioPortal. **(B)** The difference of mutation numbers between high-risk and low-risk groups. **(C,D)** Univariate Cox regression hazard analysis **(C)** and Multivariate Cox regression model **(D)** for top ten mutated genes in LGG. **(E,F)** The association of mutation score with the overall survival **(E)** and the ERG risk signature in LGG patients **(F)**. **(G)** Calibration plots for the nomogram. **(H)** Prognostic nomogram for LGG patients. **P < 0.01.

In addition to age and grade, genetic mutations are also tightly associated with brain cancer development and malignant progression (Barnholtz-Sloan et al., 2008; Weller et al., 2015). In LGG, the ten genes (*IDH1*, *CIC*, *FUBP1*, *NOTCH1*, *MUC16*, *TP53*, *ATRX*, *PIK3C1*, *TTN*, and *NF1*) with the highest mutation rates in the LGG patients obtained from cBioPortal were selected for further study (Figure 7A). Interestingly, as illustrated in Figure 7B, we found that the gene mutation status of these ten genes was closely associated with the risk score of ERGs, indicating that the gene mutation status might act as an OS predictor in LGG patients. Further univariate Cox proportional hazards regression analysis suggested that *PIK3C1* and *NF1* mutations were two hazardous factors, whereas *IDH1* mutation was an advantageous factor for the survival of LGG patients (Figure 7C). To better integrate the ten gene mutation status with the OS of LGG patients, we assigned a score of 1 to a gene with mutations while assigning a score of 0 to a gene without any mutations in an LGG sample and then perform multivariate Cox regression analysis. Finally, a mutation signature for the OS of LGG patients was established, which was composed of *TTN*, *PIK3CA*, *NF1*, and *IDH1* (Figure 7D). The mutation score to predict OS for each patient was calculated as follows: Mutation score = 0.88 × (multiply) mutation status (0 or 1) of TTN +1.12 × mutation status of *PIK3CA* +1.33 × mutation status of NF1-0.6 × mutation status of *IDH1*. Based on the mutation score, 505 LGG patients from the TCGA cohort were stratified into low and high score groups. Further, Kaplan–Meier survival analysis showed that the OS time in the low mutation score group was much longer than the high mutation score group (Figure 7E), which indicated the high mutation score was hazardous for extending the survival of LGG patients. Therefore, it could be expected that a high mutation score was positively correlated with the ERG risk score (*P* < 0.01; Figure 7F).

To provide clinicians with a quantitative approach for predicting LGG survival, a nomogram was developed to predict the probability of 3- and 5-year OS based on the 29-endocytosis-related-gene signature, age, and mutation score, which were significant factors for OS after multivariate Cox analysis (Supplementary Figure 2). As shown in Figure 7G, each factor was assigned a score in proportion to its risk contribution to survival. The calibration curves showed good consistency between actual and predicted survival, especially 3- and 5-year survival (Figure 7H).

## Discussion

Much evidence has indicated the implications of endocytic pathways for cancer progression. Dysregulated endocytosis, caused by altered expression levels of endocytic machinery components, is regarded as an emerging feature of cancer cells; however, prognostic studies of differential endocytosis-related genes (DERGs) in cancer are lacking. Here, we use LGG as a model to investigate their predictive power to prognose the survival of patients with tumors.

This study mined the mRNA expression of 676 ERGs in the TCGA-LGG cohort. Then, 62 genes were identified to be differentially expressed between LGG and nontumor brain tissues. All of these genes were found to be involved in endocytic signaling by GO term analysis. Among these genes, *EGFR* was further revealed as the hub gene. EGFR, a tyrosine kinase receptor, is highly expressed in many cancers, such as glioblastoma, colorectal cancer, and lung cancer (Yarden and Pines, 2012). EGFR-activating mutations are involved in cancer development and resistance to cancer therapies, such as EGFR inhibitor treatment and chemotherapy (Juchum et al., 2015; Sigismund et al., 2018). In LGG patients, we also observed high expression of *EGFR* in tumor tissues compared to the corresponding normal brain tissues. Despite its relative prevalence in high-grade gliomas such as GMB, *EGFRvIII*, a constitutively activating mutation with intragenic deletion of exons 2 to 7, is not often found in LGG (Newman et al., 2017; Huang et al., 2018); however, some oncogenic mutations of *EGFR*, such as A289V, R108K, and G598V, are found in LGG patients. In addition, the expression of mutated *EGFR* appears to be higher than that of wild-type *EGFR* in LGG patients; moreover, the OS of LGG patients harboring mutated *EGFR* is much shorter than that of patients with wild-type *EGFR*. During endocytosis, EGFR can upregulate RAB5 activity by inhibiting the activity of its GAP protein, RN-tre (Lanzetti et al., 2000). RAB5 is the primary regulator of early-stage endosomal trafficking, and its high activity is required for the homotypic fusion and subsequent maturation of endosomes *in vitro*. Since the formation of mature endosomes is necessary for the transduction of signals and transport of materials from the extracellular space to the intracellular environment (Murphy et al., 2009; Scita and Di Fiore, 2010), activated EGFR signaling caused by its high expression or constitutively activating mutation is expected to accelerate the endocytic process, which provides survival signals or nutrition for the growth and proliferation of cancer cells.

Next, we established a prognostic signature with 29 genes for predicting survival in the LGG cohorts. The 29 proteins are involved in different stages of the endocytosis pathway. For example, APLP1, DNM1, and PAK1 regulate the endocytic uptake of extracellular materials (Gammie et al., 1995; Neumann et al., 2006; Karjalainen et al., 2008; Grassart et al., 2010); INPP5F, CMTM3, RIN1, and RIN3 participate in early endosome maturation (Tall et al., 2001; Kajiho et al., 2003; Yoshikawa et al., 2008; Galvis et al., 2009; Yuan et al., 2017; Taefehshokr et al., 2021); CLIC4, CTSS, LAPTM4B, SYTL4, TMEM106B, S100A9, and TOM1L1 maintain the normal function of late endosomes (Munger et al., 1995; Puertollano, 2005; Ostrowski et al., 2010; Brady et al., 2013; Blom et al., 2015; Tsai et al., 2015; Hsu et al., 2019); NSG1, RAB11FIP4, RAB15, S100A10, RAB13, and RAP2A might mediate endosome recycling back to the cell surface (Zuk and Elferink, 2000; Wallace et al., 2002; Zobiack et al., 2003; Morimoto et al., 2005; Taguchi and Misaki, 2011; Parkinson and Hanley, 2018); GOLIM4, RAB29, SGSM1, and ARL4A are involved in endosome to Golgi trafficking (Natarajan and Linstedt, 2004; Lin et al., 2011; Nottingham et al., 2012; Inoshita and Imai, 2015); CORO1C and KIF5A regulate endosome fission and endosome transport in the cytosol, respectively, (Schmidt et al., 2009; Hoyer et al., 2018); VAV2 and ZFYVE28 are able to divert EGFR endosomal trafficking and lysosomal degradation, although the detailed molecular mechanisms are elusive (Mosesson et al., 2009; Thalappilly et al., 2010). The risk score was calculated for each patient based on the mRNA expression levels and risk coefficients of the 29 selected genes. The risk scores meaningfully classified patient outcomes in LGG cohorts. More particularly, the prognostic power of the 29-gene signature was verified in another LGG cohort. ROC curve analysis indicated that the risk score is a better predictor than other clinical characteristics, e.g., grade and age. Collectively, these results suggest that our gene signature could provide an accurate index for predicting LGG prognosis.

It is well known that gene mutation status plays an important role in oncogenesis and patient prognosis, indicating the potential application of characteristic gene signatures in cancer diagnosis and prognosis. For example, *BRCA* mutations are associated with unfavorable prognosis in breast cancer patients (Brekelmans et al., 2006; Zhong et al., 2015); in addition, a higher *MUC16* gene mutation rate indicates a more favorable prognosis in patients with stomach adenocarcinomas (Li et al., 2018). After analyzing the top ten mutated genes in LGG, we found that mutation of the *IDH1* had a favorable effect for LGG patients, whereas *PIK3CA* or *NF1* mutation could lead to an adverse impact on the survival of these patients. To better correlate the gene mutation information with the OS, we performed the multivariate Cox regression. It was found that the mutation status of *TTN*, *PIK3CA*, *NF1*, and *IDH1* could be used to establish a model predict the OS in LGG patients. IDH1 is a dimeric cytosolic NADP-dependent isocitrate dehydrogenase that catalyzes the decarboxylation of isocitrate into alpha-ketoglutarate (Al-Khallaf, 2017). Consistent with our results, *IDH1/2* mutations have been shown to be factors indicating a favorable prognosis in all types of gliomas (Yan et al., 2009; Cancer Genome Atlas Research Network et al., 2015; Aibaidula et al., 2017). *TNN* was the most frequently mutated gene across different cancer types, and its mutation frequency could act as an independent marker for tumor mutation burden (TMB) in multiple cancer types (Oh et al., 2020). The clinical outcome of *TNN* mutation in different cancer types is conflicted, indicating the role of mutated *TNN* in cancer is context-based (Cheng et al., 2019; Jia et al., 2019). *PIK3CA* encodes p110α, the catalytic subunit of PI3K; the mutation of *PIK3CA* stimulates the PI3K-AKT signaling pathway and promotes cell growth in various cancers (Madsen et al., 2018). NF1 acts as a tumor suppressor through inactivating Ras activity; therefore, when *NF1* is mutated, Ras-related signal pathways, e.g., MEK–ERK, and PI3K-AKT pathways, are hyperactivated, which results in poor clinical outcomes for cancer patients (Malone et al., 2014; Redig et al., 2016). After integration of the *TTN*, *PIK3CA*, *NF1*, and *IDH1* mutation status with age and the ERG risk score, a nomogram was developed and exhibited excellent performance, especially for predicting the 3- and 5-year survival probabilities of LGG patients.

In short, we first developed an ERGs expression model that could act as an independent prognostic indicator for LGG. A nomogram developed based on the gene signature and clinicopathological features accurately predicted the 3- and 5-year survival probabilities for individual LGG patients. Our findings suggest that the 29-endocytosis-gene signature may help facilitate personalized and precision medicine in clinical practice.

## Data Availability Statement

The original contributions presented in the study are included in the article/Supplementary Material, further inquiries can be directed to the corresponding author/s.

## Author Contributions

GW and DW contributed to the conception and design of the study. DW organized the database and wrote the first draft of the manuscript. DW and SL performed the statistical analysis. DW, SL, and GW wrote the manuscript. All authors contributed to manuscript revision, read, and approved the submitted version.

## Conflict of Interest

The authors declare that the research was conducted in the absence of any commercial or financial relationships that could be construed as a potential conflict of interest.

## Publisher’s Note

All claims expressed in this article are solely those of the authors and do not necessarily represent those of their affiliated organizations, or those of the publisher, the editors and the reviewers. Any product that may be evaluated in this article, or claim that may be made by its manufacturer, is not guaranteed or endorsed by the publisher.

## Footnotes

1
https://www.genecards.org/

2
https://toil.xenahubs.net

3
http://www.cgga.org.cn/

4
http://string-db.org

5
https://cytoscape.org/

6
http://software.broadinstitute.org/gsea/

7
https://gdc.xenahubs.net

8
http://www.cbioportal.org/

9
https://github.com/daweiwong/expert-spork

## Supplementary Material

Supplementary Figure 1 Specific GO and KEGG enriched by the DEERGs. **(A)** Differentially, over-, under-, and non-differentially expressed ERG genes in LGG were performed GO analysis, after which the common GO terms obtained from another three gene lists were removed from the GO results of DEERGs. **(B)** Differentially, over-, under-, and non-differentially expressed ERG genes in LGG were performed KEGG analysis, after which the common pathways obtained from another three gene lists were removed from the KEGG results of DEERGs.

Supplementary Figure 2 Multivariate regression analysis of risk score and mutation score with different clinical parameters.

Supplementary Table 1 The list of endocytosis-related genes (ERGs).

Supplementary Table 2 GO analysis of the DEERGs.

Supplementary Table 3 KEGG analysis of the DEERGs.

Supplementary Table 4 KEGG pathways significantly enriched in the high-risk score LGG patients via GSEA analysis.

## Abbreviations

LGG: Low-grade glioma
ERG: endocytosis-related gene
TCGA: the cancer genome atlas
GBM: glioblastoma multiforme
GO: gene ontology
KEGG: Kyoto encyclopedia of genes and genomes
GTEx: the genotype-tissue expression database.
