# Supplementary figures and images for: Establishment of an Endocytosis-Related Prognostic Signature for Patients With Low-Grade Glioma
Source: Front Genet. 2021 Sep 6;12:709666. doi: 10.3389/fgene.2021.709666 (PMC8450508; doi:10.3389/fgene.2021.709666)

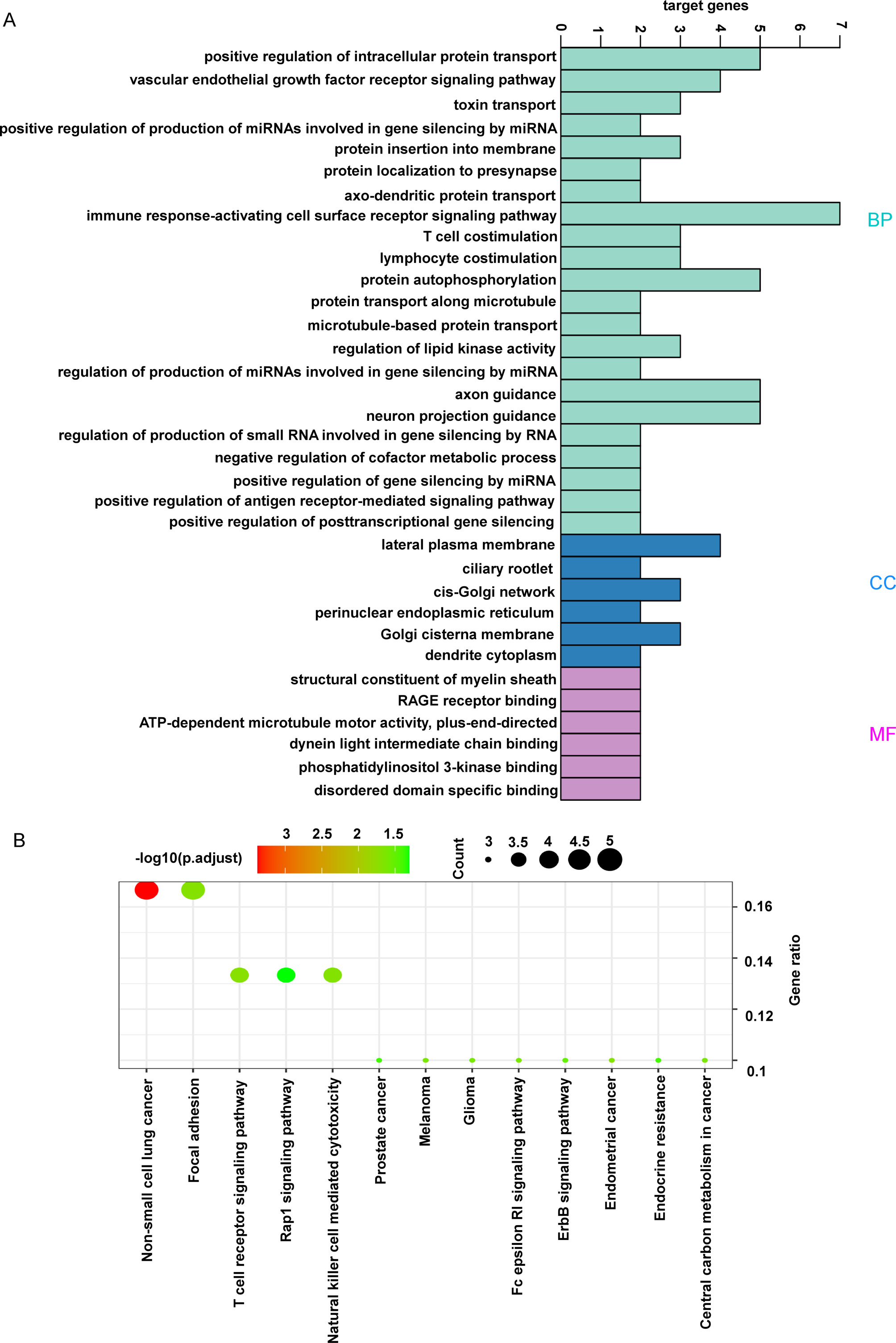

Supplement: Supplementary Figure 1 — Specific GO and KEGG enriched by the DEERGs. (A) Differentially, over-, under-, and non-differentially expressed ERG genes in LGG were performed GO analysis, after which the common GO terms obtained from another three gene lists were removed from the GO results of DEERGs. (B) Differentially, over-, under-, and non-differentially expressed ERG genes in LGG were performed KEGG analysis, after which the common pathways obtained from another three gene lists were removed from the KEGG results of DEERGs. [file Image_1.TIF]

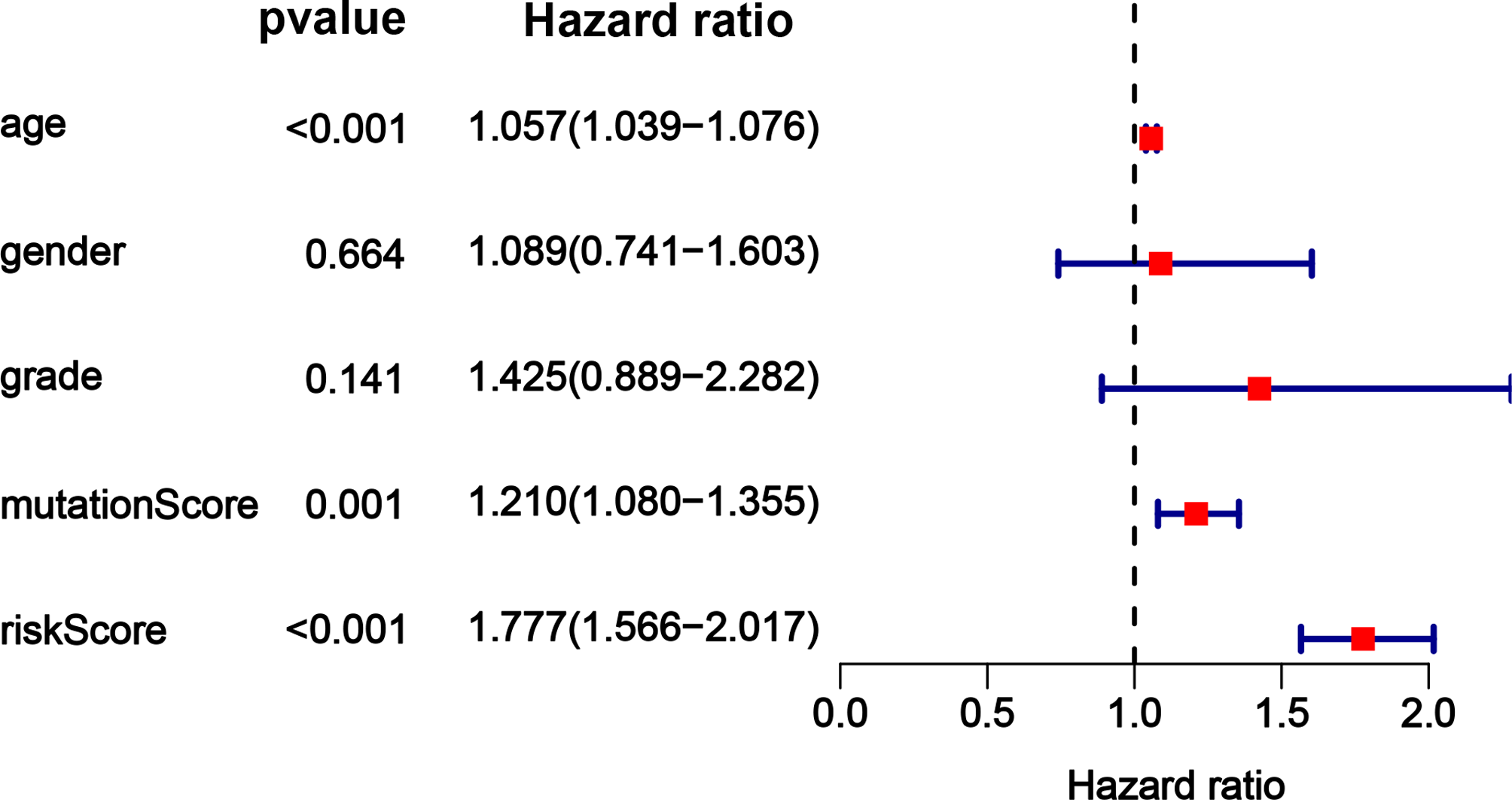

Supplement: Supplementary Figure 2 — Multivariate regression analysis of risk score and mutation score with different clinical parameters. [file Image_2.TIF]
